# Nicotine-induced survival signaling in lung cancer cells is dependent on their p53 status while its down-regulation by curcumin is independent

**DOI:** 10.1186/1476-4598-9-220

**Published:** 2010-08-20

**Authors:** Vineshkumar T Puliyappadamba, Vino T Cheriyan, Arun Kumar T Thulasidasan, Smitha V Bava, Balachandran S Vinod, Priya R Prabhu, Ranji Varghese, Arathy Bevin, Shalini Venugopal, Ruby John Anto

**Affiliations:** 1 Integrated Cancer Research Program, Division of Cancer Research, Rajiv Gandhi Centre for Biotechnology, Thiruvananthapuram, 695014, Kerala, India

## Abstract

**Background:**

Lung cancer is the most lethal cancer and almost 90% of lung cancer is due to cigarette smoking. Even though nicotine, one of the major ingredients of cigarette smoke and the causative agent for addiction, is not a carcinogen by itself, several investigators have shown that nicotine can induce cell proliferation and angiogenesis. We observed that the proliferative index of nicotine is different in the lung cancer cell lines H1299 (p53-/-) and A549 (p53+/+) which indicates that the mode of up-regulation of survival signals by nicotine might be different in cells with and without p53.

**Results:**

While low concentrations of nicotine induced activation of NF-κB, Akt, Bcl2, MAPKs, AP1 and IAPs in H1299, it failed to induce NF-κB in A549, and compared to H1299, almost 100 times higher concentration of nicotine was required to induce all other survival signals in A549. Transfection of WT-p53 and DN-p53 in H1299 and A549 respectively, reversed the mode of activation of survival signals. Curcumin down-regulated all the survival signals induced by nicotine in both the cells, irrespective of their p53 status. The hypothesis was confirmed when lower concentrations of nicotine induced NF-κB in two more lung cancer cells, Hop-92 and NCI-H522 with mutant p53 status. Silencing of p53 in A549 using siRNA made the cells susceptible to nicotine-induced NF-κB nuclear translocation as in A549 DN-p53 cells.

**Conclusions:**

The present study reveals a detrimental role of nicotine especially in lung cancer patients with impaired p53 status and identifies curcumin as a potential chemopreventive.

## Background

Lung cancer is the most lethal of all cancers worldwide with a dismal prognosis and 5 year survival rate of < 15%. Reports indicate that p53 alterations are the most common genetic events in lung cancer development and 50-60% of non-small cell lung cancers (NSCLC) and 90% of small cell lung cancers (SCLC) contain p53 mutations [1]. The major reason for lung cancer incidence is tobacco smoke, which contains nicotine. There is no evidence indicating that nicotine is a carcinogen by itself [2]; nevertheless, it has been demonstrated that nicotine promotes the growth of cancer cell populations in the lungs [3] by stimulating cell proliferation and angiogenesis [4,5]. Chronic exposure to nicotine causes mitogenic stimulus and leads to the activation of growth-promoting and antiapoptotic signaling pathways such as PI3K-Akt/mTOR, NF-κB, COX-2, Bcl2, MAPKs etc [6-10]. It has also been shown to induce secretion of several growth factors and upregulate the calpain family of proteins in lung cancer cells leading to the activation of Raf/MAPK/ERK pathway [11-13]. Moreover, reports indicate that nicotine inhibits apoptosis induced by various stimuli including chemotherapeutic agents in lung cancer cells where survivin and XIAP are suggested to play a key role [14].

Curcumin, a polyphenol isolated from *Curcuma longa*, has been extensively investigated for its cancer chemopreventive and chemotherapeutic properties, which are attributed mainly to its antioxidant and anti-inflammatory potential [15,16]. Curcumin, with its potent anti-inflammatory property, is expected to exert chemopreventive effects on carcinogenesis and has been shown to modulate numerous transcription factors, protein kinases etc. that have been linked to the pathophysiology of cancer [17]. It up-regulates several apoptosis inducing genes such as p53 and p21 [17] and down-regulates pro-survival genes such as NF-κB, Akt, Bcl2 and Cyclin D1 [17,18] induced by various stimulants. This has been suggested to be the reason for its chemosensitizing efficacy [17,19]. Despite the bioavailability of curcumin being very low, studies indicate that even at a physiologically achievable concentration, curcumin is sufficient for its chemopreventive and chemotherapeutic activity [20].

p53, which regulates many cellular activities including cell cycle arrest and apoptosis, is the most commonly mutated gene in human cancers [21]. It has been well documented that absence of functional p53 in cells render them resistant to chemotherapy, and restoration of p53 lessen the tumor occurrence [22]. One major function of p53 is to control DNA replication by inducing p21 protein, which promotes cell cycle arrest by modifying the phosphorylation of the cyclin-dependent kinases [23], although regulation of p21 expression independent of p53 also has been reported [24]. Activation of the p53-p21 pathway and induction of both p53 and p21 are often reported in response to DNA damaging agents including nicotine [25].

The nuclear transcription factor NF-κB is an important survival signal which induces the expressions of diverse target genes including COX-2, Cyclin D1 and XIAP that promote cell proliferation, regulate apoptosis and stimulate invasion and metastasis [9,26]. Studies indicate that simultaneous inhibition of p53 and NF-κB transcriptional activities decide the fate of the cell [27,28]. Previous studies have also demonstrated that nicotine activates signal transduction pathways relevant to carcinogenesis through the activations of MAPK and PI3-K/Akt pathways [6,7].

In the present study we have tried to correlate the effect of nicotine to the p53 status of lung cancer cells, especially NSCLC cell lines of adenocarcinoma or large cell carcinoma origin. We selected these cells for our study because cigarette smoking is a major risk factor in the development of NSCLC, which accounts for 80% of all lung cancers [14]. We report in this study that the induction of survival signals in lung cancer cells by nicotine is dependent on the p53 status of the cells and identify p53 status as an important predictor of lung cancer progression. Additionally, we have attempted in delineating the signaling cross-talk mechanism orchestrating p53-dependent regulation and expression of survival signals such as NF-κB, AP-1, Akt, Bcl2 etc., in the presence and absence of p53 expression. Our findings underscore that p53 plays an essential role in regulating survival signals, especially NF-κB, in response to nicotine. We also present data supporting our contention that curcumin down-regulates all the survival signals induced by nicotine, independent of the p53 status of the cell.

## Results

### Nicotine induces more proliferation in lung cancer cells lacking p53, which is down-regulated by curcumin

**Figure 1:**
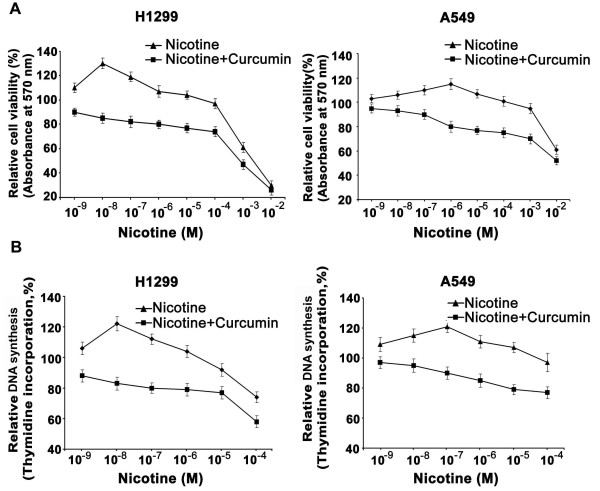
**Nicotine induces more proliferation in H1299 than in A549, which is down regulated by curcumin**. (A-B) The cells were treated with nicotine and/or curcumin for 72 h and 24 h respectively for MTT assay and thymidine incorporation assay. Tritiated thymidine (0.5 μCi/well) was added 6 h before the completion of incubation. All error bars indicate standard deviation between three independent experiments.

Even though nicotine-induced proliferation of lung cancer cells has been reported [4], our findings show for the first time that, among the lung cancer cell lines, nicotine induces more proliferation in H1299 lacking p53 especially at lower concentrations (10^-9^M -10^-7^M) than A549 with active p53 (maximum at 10^-7^M -10^-6^M). Of interest, pre-treatment with a non-toxic concentration of curcumin significantly reduced (p < 0.001) the viability of both lung cancer cells (Fig 1. A-B).

### Nicotine induces NF-κB in H1299 cells but not in A549 cells

**Figure 2:**
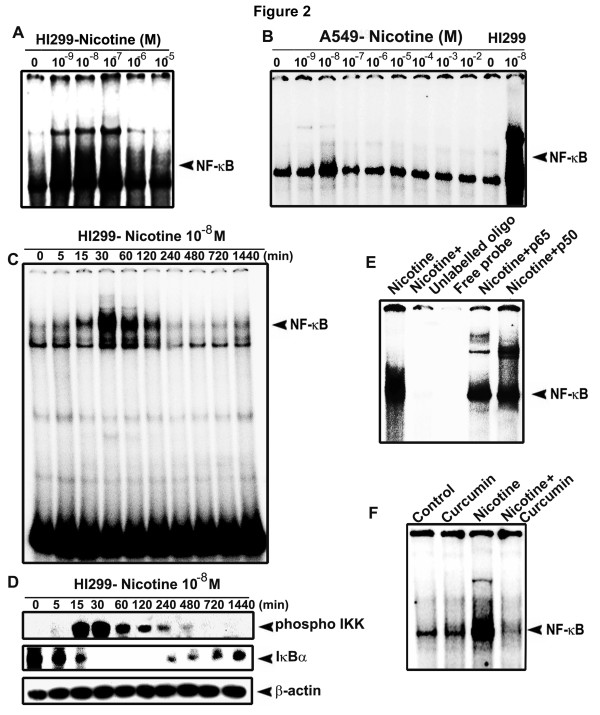
**Nicotine activates NF-κB in H1299, but not in A549**. (A-B) Nuclear extracts were prepared from the cells treated with indicated concentrations of nicotine for 30 min and NF-κB nuclear translocation was assessed by EMSA. (C) H1299 cells were treated with 10^-8 ^M nicotine for different time intervals and EMSA was done. (D) The cytosolic extracts of the time kinetic study of NF-κB activation were Western blotted against the respective antibodies. (E) The nuclear extract of nicotine-treated H1299 cells were incubated with p50 and p65 antibodies or unlabelled oligo and EMSA was conducted to detect supershift and cold competition respectively. (F) H1299 cells were treated with curcumin and then with nicotine for 30 min and EMSA was done. All blots and EMSAs are representative samples of three independent experiments.

Nicotine has been shown to activate NF-κB in cancer cells of various origins, including lung [3,8]. We also have reported earlier that cigarette smoke induces strong activation of NF-κB in H1299 cells [29]. In the present study, H1299 and A549 cells were exposed to various concentrations of nicotine and the status of nuclear translocation of NF-κB was studied. Interestingly, nicotine could activate NF-κB only in H1299 cells (Fig. 2A) while it failed to induce a significant activation of NF-κB in A549 cells at any of the concentrations studied (Fig. 2B). In H1299 cells, lower concentrations (10^-9^M-10^-7^M) of nicotine induced maximum activation of NF-κB and a time kinetics study using 10^-8 ^M nicotine showed maximum activation at 30 min of exposure to nicotine (Fig. 2C). In congruence, the phosphorylation pattern of IKK and degradation pattern of IκBα (Fig. 2D) correlated with that of the NF-κB activation, attesting that nicotine induces NF-κB through the classic pathway. The specificity of the NF-κB band was verified by super shift assay and by cold competition (Fig. 2E). The activation of NF-κB induced by nicotine in H1299 cells was completely abolished by treatment with 10 μM curcumin (Fig. 2F).

### Nicotine-induced phosphorylation of Akt and over expression of COX-2 and Cyclin D1 in lung cancer cells are inhibited by curcumin

**Figure 3:**
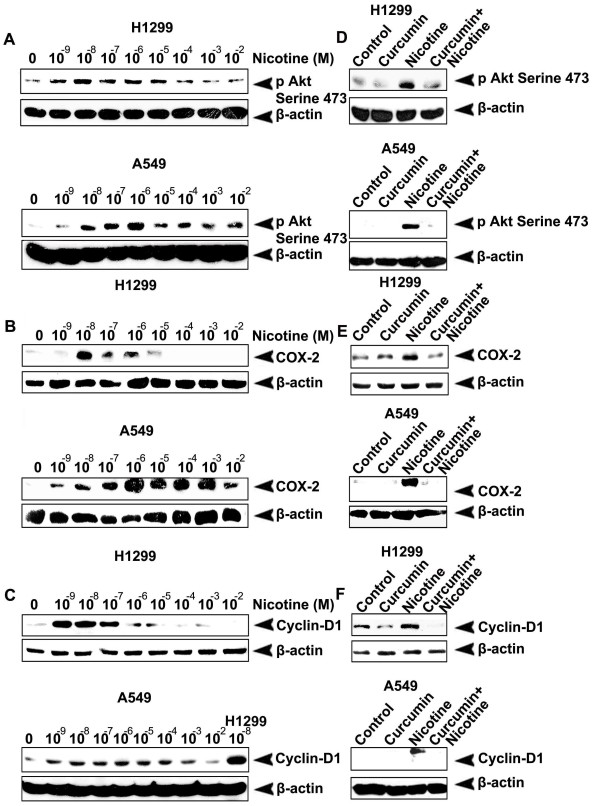
**Curcumin inhibits nicotine-induced phosphorylation of Akt and over expression of COX-2 and Cyclin D1**. (A-C) The cells were treated with various concentrations of nicotine (30 min for phospho-Akt and 24 h for COX-2 and Cyclin-D1) and whole cell lysates prepared were blotted against phospho-Akt, COX-2 and Cyclin-D1 antibodies. (D-F) The cells were treated with curcumin and then with nicotine (10^-8 ^M in H1299 and 10^-6 ^M in A549) and whole cell lysates were blotted as in (A-C). All blots are representative samples of three independent experiments.

We compared the effect of nicotine in A549 and H1299 cells in inducing pro-survival molecular entities-Akt, COX-2 and Cyclin D1, which generally get activated in response to NF-κB activation. Though nicotine was ineffective in inducing NF-κB in A549 cells, it effectively induced Akt, COX-2 and Cyclin D1 in both the cells (Fig. 3A-C) indicating that nicotine activates these pro-survival signaling molecules independent of NF-κB. However, there was a marked difference in the expression pattern of all these survival signals between the investigated cell lines. It was noticed that, while nicotine induces activation of these survival molecules in the range of 10^-9^M - 10^-7^M in H1299 cells, a 10-100 fold high concentration of nicotine was needed to induce the same effect in A549 (Fig. 3A-C). Despite studies reporting the interdependence and cross-talk between Akt and NF-κB [26], phosphorylation of Akt by PI3K pathway independent of NF-κB is also well evidenced [30-33]. Similarly, while several reports strongly correlate the involvement of NF-κB in Cyclin D1 regulation [30], there are reports which clearly demonstrate the transcription of Cyclin D1, independent of NF-κB, in cell lines including A549 [32,4]. The protein level expression of Cyclin D1 in A549 was low compared to that in H1299 cells at all the concentrations studied (Fig. 3C). We infer from these results that nicotine-induced expression of these survival signals was severely compromised in the presence of curcumin in both the investigated cell lines (Fig. 3D-F) and hence we draw attention to the protective role of curcumin against survival and proliferative signals induced by nicotine.

### Curcumin prevents the over-expression of IAPs and Bcl2 by nicotine

**Figure 4:**
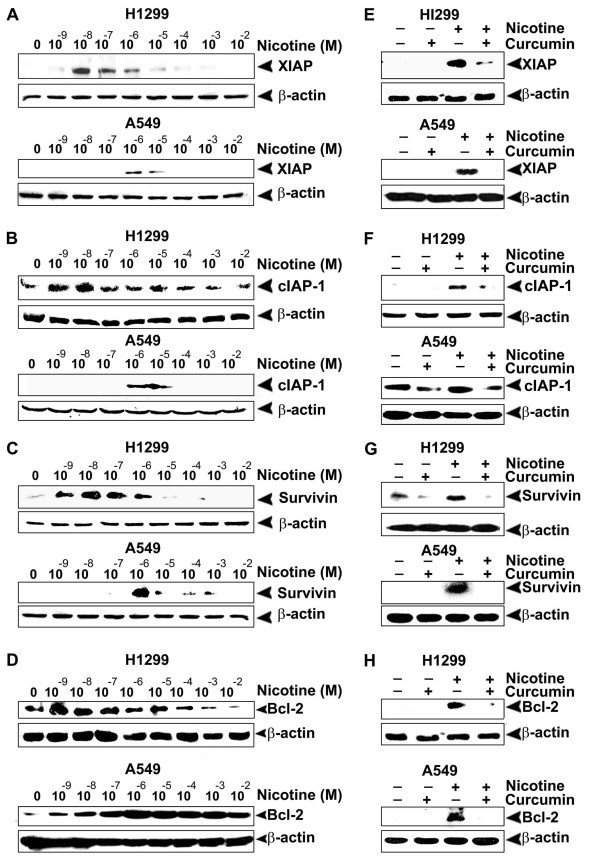
**Nicotine-induced over expression of IAPs and Bcl2 is inhibited by curcumin**. (A-D) The cells were treated with nicotine for 24 h and whole cell lysates were Western blotted using antibodies against XIAP, cIAP-1 survivin and Bcl2. (E-H) The cells were treated with curcumin and then with nicotine (10^-8^M for H1299 and 10^-6 ^M for A549) and whole cell lysates were blotted as in (A-D). All blots are representative samples of three independent experiments.

The IAP family of proteins involved in apoptosis regulation is generally activated in response to NF-κB activation [33]. Consistent with previous reports indicating that 1 μM nicotine induces the IAP family members, XIAP and survivin [14], we also observed the over-expression of XIAP and survivin in A549 and H1299 cell lines, albeit difference in the concentration of nicotine for effective stimulation of these proteins. However, we observed robust expression of cIAP-1 too in both the cell lines by nicotine (Fig. 4A-C), though Dasgupta *et al *[14] did not observe it in A549 cells. It was inferred that while nicotine induces activation of these survival signals in the range 10^-9 ^M-10^-7 ^M in H1299 cells, it induces the same level of activation in A549 cells at 10^-6 ^M-10^-4 ^M range (Fig. 4A-C). Additionally, because Bcl2 is considered as a general inhibitor of apoptosis, we evaluated the role of nicotine in its induction above basal level and tested the effect of curcumin in conferring chemoprotection. As in the case of other survival signals, a 10-100 fold higher concentration of nicotine was necessary to induce Bcl2 in A549 cells, compared to H1299 cells (Fig. 4D) although, curcumin abolished it more or less completely in both the cell lines. Results obtained thus far indicate that in the investigated lung cancer cell lines, curcumin completely inhibits the over-expression of these survival signals (Fig. 4E-H) independent of the p53 status and the concentration of nicotine, strongly suggesting a preventive action of curcumin against nicotine-induced promotion and progression of lung cancer through up-regulation of antiapoptotic molecules.

### Nicotine-induced phosphorylation of MAPKs and nuclear translocation of AP-1 in lung cancer cells are down-regulated by curcumin

**Figure 5:**
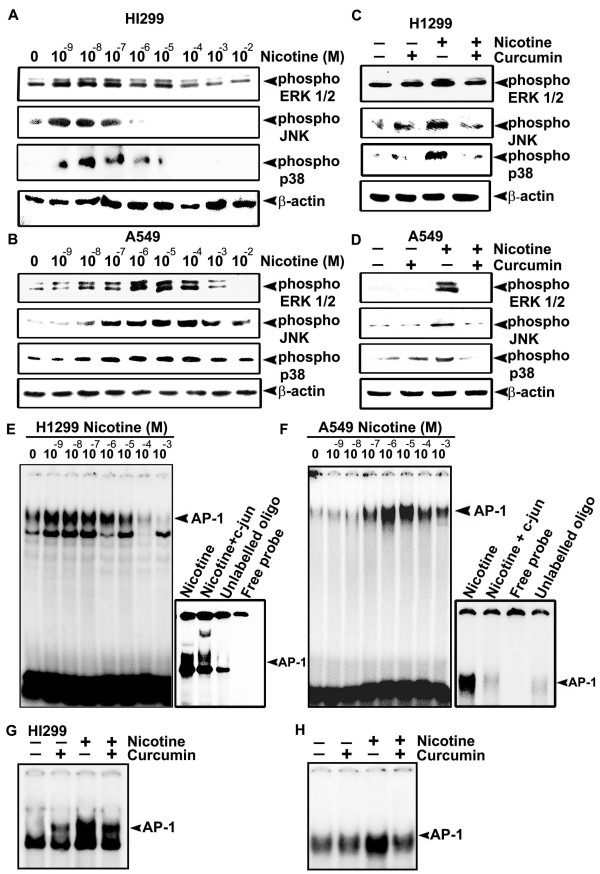
**Curcumin downregulates nicotine-induced phosphorylation of MAPKs and nuclear translocation of AP-1**. (A-B) The cells were treated with nicotine (10^-9^-10^-2^M) for 30 min and whole cell lysates were blotted against respective phospho specific antibodies. (C-D) The cells were treated with curcumin and then with nicotine (10^-8^M for H1299 and 10^-6 ^M for A549) and whole cell lysates were blotted as in (A-B). (E-F) Nuclear extracts were prepared from the cells treated with nicotine (10^-9^-10^-2^M) for 30 min. and AP-1 activation was assessed by EMSA. Super shift and cold competition was done by incubating the nuclear extract with c-jun antibody and unlabelled oligo respectively. (G-H) The cells were treated with curcumin and then with nicotine (10^-8^M for H1299 and 10^-6 ^M for A549) and EMSA was done using nuclear extracts. All blots and EMSAs are representative samples of three independent experiments.

Over-expression of cyclin D1 and IAPs by nicotine even in the absence of NF-κB led us to speculate the involvement of other pathways regulating the expression of these proteins. Involvement of the MAPK pathway [7] and the induction of cyclin D1 through the MAPK-AP-1 pathway [34] in nicotine-induced cell signaling is well evidenced. Induction of IAPs by MAPKs independent of NF-κB has also been reported [35]. We therefore performed a time kinetic study up to 120 min to investigate the functional role of MAPK in H1299 and A549 cells, by analyzing their phosphorylation status in the presence of nicotine and found that, nicotine induces maximum phosphorylation of all the MAPKs at 30 min (data not shown). Dose response study revealed that all the three MAPKs are phosphorylated in both the cell lines, being maximally activated at 10^-8^M concentration of nicotine in H1299 cells (Fig. 5A) and at 10^-6 ^M in A549 cells (Fig. 5B). However, curcumin treatment (10 μM) inhibited the phosphorylation of these MAPKs by nicotine in both the cells (Fig. 5C-D).

Regulation of MAPK pathway by nicotine through p53 has already been reported [25,36]. We therefore assessed whether p53 has any regulatory role in the activation of AP-1, which gets translocated to the nucleus in response to the phosphorylation of MAPKs since, up-regulation of AP-1 activity by nicotine has been reported in the literature [37]. Interestingly, though nicotine failed to activate NF-κB in A549 cells, it activated AP-1 very efficiently in both H1299 and A549 cells (Fig. 5E-F) though the pattern of nuclear translocation of AP-1 was in parallel with the phosphorylation pattern of MAPKs in the respective cell lines. As in the case of MAPKs, curcumin treatment down regulated this activation almost completely in both the cells (Fig. 5G-H).

### Introduction and inactivation of p53 in H1299 and A549 cells respectively, bring about reciprocal effect on the activation pattern of NF-κB, MAPKs and AP-1 in both the cells

**Figure 6:**
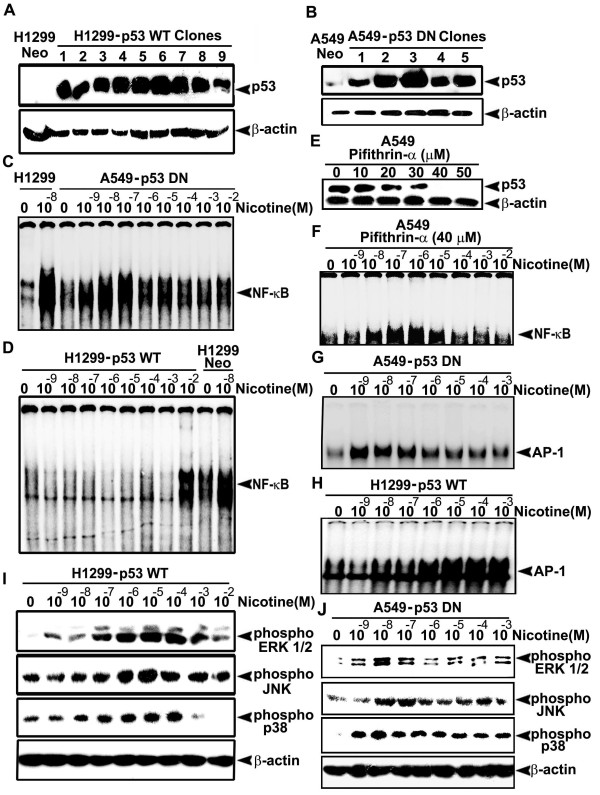
**Over-expression and silencing of p53 produce reciprocal effect on the activation pattern of NF-κB, MAPKs and AP-1**. (A-B) H1299 and A549 cells were transfected with pcDNA3-p53 WT and pcDNA3-p53 DN constructs respectively and the stable clones selected were lysed and Western blotted against p53 antibody. (C-D) H1299-p53 WT and A549-p53 DN cells were treated with nicotine (10^-9^-10^-2^M) for 30 min, and EMSA was done using nuclear extracts. (E) A549 cells were treated with different concentrations of pifithrin-α (10-50 μM) for 4 h and the whole cell lyasates prepared were Western blotted against p53 antibody. (F) A549 cells were treated with 40 μM pifithrin-α for 4 h followed by nicotine (10^-9^-10^-2^M) for 30 min and EMSA was done. (G-H) H1299-p53 WT and A549-p53 DN cells were treated with nicotine (10^-9^-10^-2^M) for 30 min, and EMSA was done. (I-J) H1299 WT-p53 and A549 DN-p53 cells were treated with nicotine (10^-9^-10^-2^M) for 30 min and whole cell lysates were Western blotted against respective phospho specific antibodies. All blots and EMSAs are representative samples of three independent experiments.

The correlation between NF-κB nuclear translocation and the p53 status of cells is controversial. Some researchers contend that p53 is needed for NF-κB activation [38,39] while, others have shown that activation of NF-κB can be induced in a p53 independent manner [40]. We noticed differences in the activation pattern of NF-κB, MAPKs and AP-1 in cells harboring p53 versus cells lacking p53. To corroborate whether this distinction is due to the p53 status of the cells, we transfected H1299 cells with p53 WT plasmid and A549 cells with p53 DN plasmids, followed by selection of the clones with maximum p53 expression for further studies. In H1299-p53 WT, clone 6 (Fig. 6A) and in A549-p53 DN, clone 3 was selected (Fig. 6B). Whereas nicotine failed to induce NF-κB nuclear translocation in A549 cells, it caused significant induction of NF-κB in A549-p53 DN cells within the concentration range of 10^-9^M - 10^-7^M similar to H1299 cells (Fig. 6C). Supporting this observation, inhibition of p53 in A549 cells using 40 μM pifithrin-α, which completely inhibits p53 (Fig. 6E) induced NF-κB nuclear translocation in A549 cells (Fig. 6F). Likewise, nicotine failed to induce NF-κB activation in H1299-p53 WT cells except at a very high concentration of 10^-2 ^M (Fig. 6D) though, it induced very strong activation of NF-κB in H1299 cells at 10^-9^-10^-7 ^M. This confirms the interdependence between NF-κB and p53. Furthermore, the mode of phosphorylation of MAPKs and the nuclear translocation of AP-1 by nicotine was also seen to be reversed in the transfected cells (Fig. 6G-J), further adding support to our hypothesis.

### Proliferative as well as clonogenic potential induced by nicotine may be regulated through up-regulation of p53 and p21, and is down-regulated by curcumin

**Figure 7:**
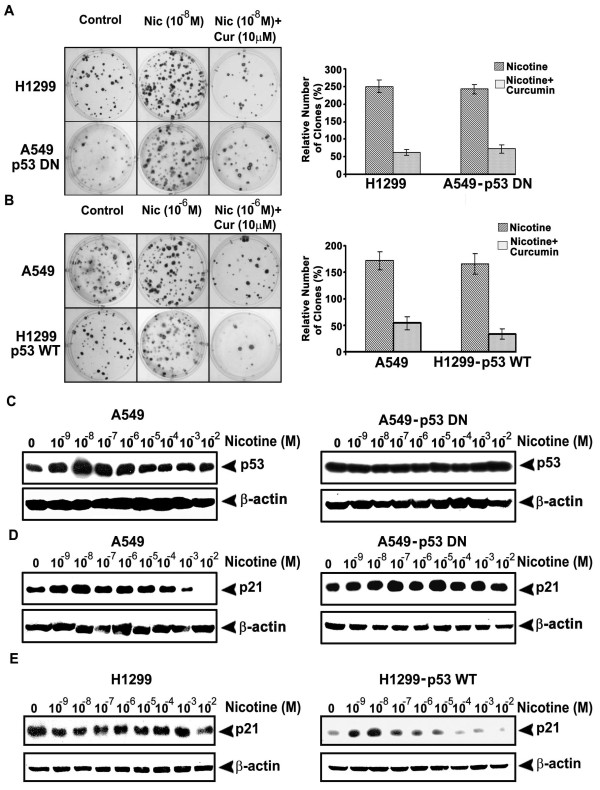
**Curcumin inhibits enhancement of clonogenicity by nicotine, which induces p53 and p21 in A549 and p21 in H1299-p53 WT**. (A-B) The cells were treated with nicotine and/or curcumin for 72 h, after which cells were split and seeded in six well plates, kept for 2 weeks and clones developed were stained and counted. (C-E) The cells were treated with nicotine (10^-9^-10^-2^M) for 24 h and whole cell lysates were Western blotted against p53 antibody. All figures are representative samples of three independent experiments.

Because of the difference in the p53 status between the investigated cells, we studied whether p53 can differentially regulate the clonogenicity by nicotine in these cells. While 10^-8^M nicotine induced maximum number of clones in H1299 cells, 10^-6^M nicotine produced maximum proliferation in A549 cells as indicated by the bigger size of the colonies formed (Fig. 7A-B). Moreover, the number and size of the clones was much higher in H1299 cells compared to A549. As expected, the number of clones by nicotine addition in A549 was escalated when p53 was shut down, whereas those produced in H1299 was significantly reduced (p < 0.005) when p53 was transfected (Fig. 7A-B). However, in both the cells, pretreatment with curcumin (10 μM, 2 h) drastically reduced the number and size of the clones induced by nicotine, clearly indicating that curcumin negatively regulates clonogenicity (Fig. 7A-B).

As part of investigation of this difference in the activation pattern of various survival signals by nicotine in cells with and without p53, we evaluated the effect of nicotine on expression levels of p53 and p21 in A549 parental and A549-p53 DN cells. Interestingly, while lower concentrations (10^-9 ^M - 10^-7 ^M) of nicotine induced over-expression of p53 and p21 in A549 cells, it failed to induce significant changes in the expression pattern of p53 and p21 in A549-p53 DN cells (Fig. 7C-D). To check whether p53 is the only molecule regulating the p21 signaling in A549 cells, we also investigated the expression status of p21 in response to nicotine in H1299 cells. We did not find any significant difference in the expression of p21 in H1299 cells (Fig. 7E), implying that p53 is the key regulator of p21 in nicotine-induced signaling events. To confirm this hypothesis, we checked the p21 status in H1299-p53 WT cells in response to nicotine. As in the case of A549 cells, a strong induction of p53 was produced by nicotine in these cells (Fig. 7E) which confirms the regulatory role of p53 in nicotine-induced p21 expression.

### Loss of function of p53 enhances nicotine-induced proliferation and NF-κB nuclear translocation in lung cancer cells

**Figure 8:**
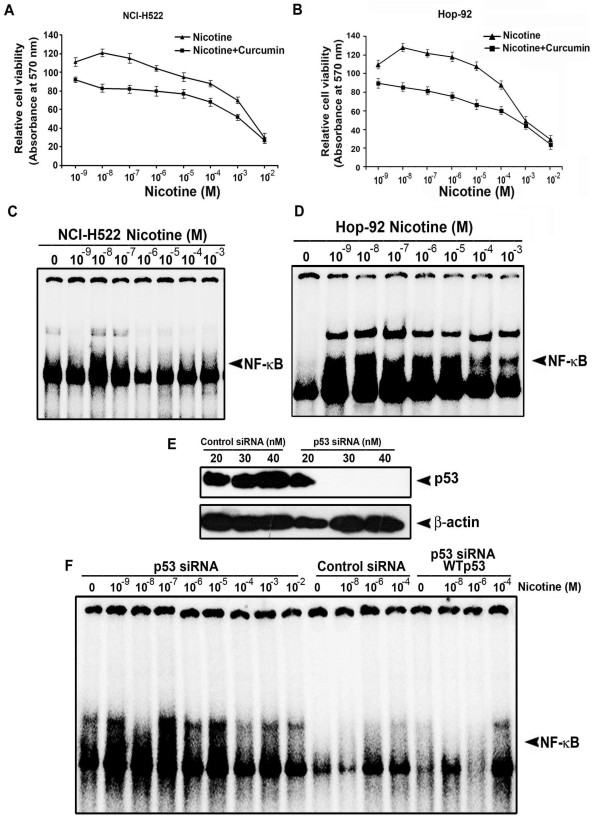
**Nicotine induces proliferation and NF-κB nuclear translocation in Hop-92, NCI-H522 and p53-silenced A549 cells**. (A-B) The cells were treated with nicotine and/or curcumin and cell viability was measured by MTT assay. All error bars indicate standard deviation between three independent experiments. (C-D) Nuclear extracts were prepared from the cells treated with indicated concentrations of nicotine for 30 min and NF-κB nuclear translocation was assessed by EMSA. (E) A549 cells were transfected with indicated concentrations of control-si RNA or p53-si RNA and after 48 h, the whole cell lysates were Western blotted against p53 antibody. (F) A549 cells were transfected with indicated concentrations of control-si RNA or p53-si RNA and after 48 h, treated with indicated concentrations of nicotine for 30 min and NF-κB nuclear translocation was assessed by EMSA. All blots and EMSAs are representative samples of three independent experiments.

We further validated our hypothesis that impaired p53 status leads to proliferation and induction of survival signals in lung cancer cells, by treating two other lung cancer cells harboring mutant p53(Hop-92 and NCI-H522) with nicotine and curcumin. We observed a significant proliferation of these cells by lower concentrations of nicotine (p < 0.001) which is prevented by curcumin pre-treatment (Fig. 8A-B). As in H1299, nicotine induced NF-κB nuclear translocation in these cells too (Fig. 8C-D). Moreover, as we observed in A549-p53 DN cells, nicotine induced NF-κB nuclear translocation when p53 was silenced in A549 cells transfected with p53 siRNA and interestingly, co-transfection of these cells with WT p53 completely inhibited the nuclear translocation of NF-κB (Fig. 8E-F) confirming the role of p53 in regulating nicotine-induced cell signaling.

## Discussion

Enormous efforts have been devoted by various research groups to identify the role of nicotine in the process of carcinogenesis by tobacco. It has been implicated as a potential risk factor in the proliferation of various cancer cells [14,41] and has been shown to induce enhanced tissue perfusion and angiogenesis [5,42]. In the present study, we have convincingly shown that nicotine induces proliferation in lung cancer cells, especially in those with impaired p53 status. It also induces an array of antiapoptotic factors in these cells. Although nicotine has been shown to induce proliferation in lung cancer cells [4], the regulatory role of p53 on nicotine-induced proliferation has not yet been addressed. We observed a significant increase in the proliferation of lung cancer cells lacking p53. This established a ground for us to evaluate the effect of p53 in the induction of various survival signals by nicotine.

The relation between NF-κB and p53 is controversial. Conceptually, over-expression of NF-κB is linked to the development and progression of various tumors [43] and inhibition of NF-κB function allegedly suppress tumorigenesis and leads to the regression of tumors. It has been documented that both NF-κB and p53 compete for the transcriptional co-activators [28]. Recent reports suggest that the presence of p53 prevents NF-κB activation though, the mechanism is not clear. However, contradicting these observations, p53-induced NF-κB activation has also been reported [44]. It has also been shown that inhibition of p53 does not inhibit NF-κB function [27].

In our present study we observed a strong activation of NF-κB by nicotine in the p53 null H1299 cell line, while no significant activation of the same was noted in the p53 proficient A549 cells, at any of the concentrations studied. Zhang *et al *[7] have reported that pre-treatment with 100 μM nicotine up-regulate both mRNA and protein level expression of NF-κB in A549. However, supporting our observation, Moodie *et al *[45] have reported that cigarette smoke condensate, in which nicotine is one of the major components (0.3 mg/cigarette), failed to induce NF-κB in A549 cells.

A cross-talk between Akt and p53 acting as cell control machinery for switching between survival and death has been reported. Akt activation has also been reported in p53 null cells [27]. In our study, 10^-9^M - 10^-8^M nicotine induced phosphorylation of Akt in H1299 cells. In contrast, a 100 fold higher concentration of nicotine was required to produce similar effects in A549. Several reports indicate interdependence between NF-κB and Akt [26]. However, Akt independent activation of NF-κB has also been reported [30]. In the present study we noted a strong phosphorylation of Akt by nicotine in A549 cells, although it failed to induce NF-κB in A549. This indicates that, nicotine-induced Akt phosphorylation is independent of NF-κB activation status of the cell.

No clear consensus between p53 and COX-2 exists in the literature. While some studies show that p53 up-regulates COX-2 [46], others show that p53 down-regulates it [47]. We observed induction of COX-2 by nicotine in A549 cells though it failed to induce NF-κB, strengthening the notion that nicotine-induced COX-2 activation in A549 cells is independent of NF-κB. Even though COX-2 is generally considered as an NF-κB dependent gene [26], there are studies which report the activation of COX-2 independent of NF-κB [48]. In parallel, we observed an NF-κB and p53 independent induction of Cyclin D1 by nicotine, though the expression profile was less in A549 cells. Cyclin D1 is also considered as an NF-κB dependent gene [49] although, recent studies implicate Cyclin D1 expression through pathways independent of NF-κB [32].

IAP family of proteins is another essential target of NF-κB [50]. The present study as well as a previous study by Dasgupta *et al *[14], have noticed over-expression of IAPs by nicotine in A549 and H1299 cells. Of interest, this is the first study reporting the difference in the activation pattern of IAPs between both these cell lines. Bcl2 is another molecule which has a key role in regulating nicotine-induced survival signaling and has often been considered as NF-κB dependent. Interestingly, we observed Bcl2 up-regulation in both the cell lines, though the pattern of up-regulation varied between cell lines. In rapport to our observation, a novel nicotine-stimulated survival pathway that involves Bcl2 phosphorylation through MAPK pathway has been reported earlier [10].

Nicotine also induces p53 [25] which, often regulates the phosphorylation pattern of MAPKs [36,51]. On the contrary, another study reports that MAPK activation occurs only in cells with functional p53 [52], indicating a reciprocal interaction between p53 signaling pathway and MAPK pathway. We observed a discrete difference in the phosphorylation pattern of MAPKs in both the cells. The involvement of p53 was further confirmed when the phosphorylation pattern was reversed when p53 was inactivated in A549 and introduced in H1299 cells.

AP-1 is a transcriptional regulator and phosphorylation of MAPKs leads to nuclear translocation of AP-1 [53]. Although NF-κB and AP-1 are regulated by different mechanisms, several studies indicate that they are activated simultaneously [54]. Nicotine induced nuclear translocation of AP-1 in both the cell lines, even though it failed to induce NF-κB in A549. However, as in the case of MAPKs, depending on the p53 status of the cell line, there was a significant change in the activation pattern of AP-1. In support of the involvement of p53 in AP1 activation, the nuclear translocation pattern of AP-1 was reversed in A549-p53DN and H1299-p53WT cells.

The ability of nicotine to enhance adherence-independent proliferation of tumor cells is well documented [55]. We observed a marked difference in the number and size of the clones between both H1299 and A549 cells treated with nicotine, which was strongly corroborating within the concentration range of nicotine at which the survival signals are activated in both the cells, implicating a regulatory role of these survival signals on nicotine-induced enhancement of clonogenic potential. As there was a drastic reduction in the number of clones on treatment with curcumin in both the cells, it is also evident that curcumin inhibits the effect of nicotine by down-regulating these survival signals. Nicotine has a high toxicity in comparison to many other alkaloids such as cocaine and higher doses of nicotine have been reported to be lethal (0.5-1.0 mg/kg for adult humans, and 10 mg for children) [56]. In our study, we also observed that all the survival signals induced by nicotine are abrogated at higher concentrations suggesting that there may be a balance between the pro-survival and anti-survival signals induced by this compound at different concentrations. However the exact concentration at which this switching over occurs is still unclear as we observed the cessation of various survival signals at different concentrations of nicotine.

Activation of almost all the survival signals investigated in this study is involved in the process of lung cancer progression. It has also been shown that p53 and p21 negatively regulate these survival signals [28,47,23]. Induction of p53 and its downstream target p21 by nicotine has been correlated to the inhibition of cell proliferation by nicotine [25]. We observed a strong induction of p53 as well as p21 by lower concentrations of nicotine in A549 cells which may be the reason for the absence of proliferative signals at this concentration in these cells. A strong induction of p21 was noted in response to nicotine in H1299 cells transfected with wild type p53 as well. But we did not observe any change in the expression of p53 in A549-p53DN cells. This is expected since p53 is non-functional in these cells, and hence cannot regulate the expression of p21. Similarly, no difference in expression of p21 in response to nicotine treatment was noted in H1299 cells as well as A549-p53DN cells, again confirming the role of p53 in regulating the nicotine-induced signaling events. Hence, we hypothesize that the induction of p53 and p21 by lower concentrations of nicotine in A549 cells may be one of the reasons why nicotine failed to induce these survival signals at these concentrations in A549, though further studies are required to confirm this hypothesis.

Our hypothesis that p53 is the key regulator of nicotine-induced survival signaling in lung cancer cells, was further authenticated in two more lung cancer cells with mutant p53 status. Lower concentrations of nicotine induced proliferation in these cells and pre-treatment with curcumin prevented it and, as in H1299, lower concentrations of nicotine induced NF-κB nuclear translocation in these cells too. In addition, when p53 was silenced in A549 cells by p53 siRNA, as in A549-p53 DN cells, nicotine induced a strong nuclear translocation of NF-κB, while co-transfection of these cells with WT p53 completely inhibited the same, substantiating the regulatory role of p53 in nicotine signaling.

The data obtained from our study is very significant because the average plasma level concentration of nicotine in a typical pack per day smoker is between 20-40 mg [57]. Studies have indicated that chemotherapeutic drugs like cisplatin, which induce p53 in cells with a wild-type p53 gene can induce apoptosis in these cells while cells with mutated p53 are unaffected [58]. Supporting this observation, Dasgupta *et al *[14] have shown that nicotine prevents the cisplatin-induced apoptosis in A549 cells with active p53. We also disclose a detrimental role of nicotine in lung cancer patients with mutant p53 status. Hence our study has proved beyond doubt that p53 has a significant role in regulating the survival signals induced by nicotine in lung cancer cells. It is also clear that curcumin down-regulates these survival signals induced by nicotine in both the cells, independent of their p53 status.

## Conclusions

Our study identifies nicotine as a potential tumor promoter, especially in people with impaired p53 status and illustrates a chemopreventive role to curcumin against nicotine-induced lung cancer progression.

## Methods

### Cell lines

H1299 cell line was a gift from Prof. BB Aggarwal, MD Anderson, Houston, A549 and NCI-H522 cells were procured from NCCS, Pune, and Hop-92 was obtained from NCI, Bethesda, MD.

### Chemicals

Antibodies against p53, IκBα, XIAP and cIAP-1 and phosphospecific antibodies against-IKK, p38, Akt and p42/44 were purchased from Cell Signalling (Beverly, MA) and those against p50, RelA, pJNK, COX-2 and Survivin were obtained from Santa Cruz Biotechnology (Santa Cruz, CA). G418 and pifithrin-α were procured from Calbiochem (San Diego, USA). Lipofectamine was procured from Invitrogen (Carlsbad, CA) and control siRNA and p53 siRNA were procured from Dharmacon, Inc (Chicago, IL). All other chemicals were purchased from Sigma Chemicals (St. Louis, MO).

### Mode of treatment

In all combination treatments 10 μM curcumin was added 2 h before nicotine (10^-8 ^M in H1299 and 10^-6 ^M in A549).

### MTT Assay

Proliferative/cytotoxic effect of nicotine and/or curcumin was determined by MTT assay as described earlier [18].

### [^3^H] Thymidine incorporation

For [^3^H] thymidine incorporation assay, cells were seeded in 96 well plates and treated with required concentrations of the drugs. After 18 h of incubation, [^3^H] thymidine was added (0.5 μCi/well) and further incubated for 6 h. Then the culture medium was removed, the cells were washed twice with phosphate-buffered saline (PBS), treated with 5% trichloroacetic acid, pelleted and supernatant was removed. Cells were then washed with ethanol and solubilized with 0.2N NaOH, contents of each well were mixed with 3 ml scintillation fluid and the radioactivity was counted using a liquid scintillation counter [59]. The experiment was repeated thrice and the error bars indicate standard deviation.

### Electrophoretic Mobility Shift Assay (EMSA)

To detect activation of NF-κB and AP-1, EMSA was done and visualized by a Phosphor Imager (Bio-Rad Personal FX). The specificity of the bands were confirmed by super shift [29].

### Isolation of Plasmids

The plasmids were isolated by the standard alkaline lysis method [60].

### Stable Transfection

H1299 Cells were transfected with pcDNA3-p53WT or the empty vector pcDNA3 while A549 cells were transfected with pcDNA3-p53 DN or the empty vector pcDNA3 using the calcium-phosphate transfection kit (Life Technologies, Inc.) according to manufacturer's protocol and selected using G418 (150 μg/ml) [61].

### siRNA Transfection

A549 cells were transiently transfected with control siRNA, p53 siRNA and/or WT-p53 plasmid using lipofectamine 2000 reagent according to manufactures protocol (Invitrogen).

### Western blot

The total protein after treatment with nicotine and/or curcumin was electroblotted as described earlier [18] and developed by Enhanced Chemiluminescence (Immobilon Western, Miliipore, Billerica, MA).

### Clonogenic assay

The cells were treated with nicotine and/or curcumin for 72 h, as in MTT assay after which the cells were split and seeded in six well plates and clonogenic assay was done as described by Franken *et al *[62].

### Statistical analysis

Statistical significance was calculated using one-way ANOVA followed by Tukey post-hoc analysis.

## Abbreviations

AP-1: Activator protein 1; Bcl2: B-cell lymphoma 2; cIAP: Cellular Inhibitor of apoptosis protein; COX2: Cyclooxygenase 2; DN: Dominant Negative; EMSA: Electrophoretic mobility shift assay; ERK: Extracellular Signal Regulated Kinase; Fig.: Figure; h: Hour; IAP: Inhibitor of apoptosis protein; IκBα: Inhibitor of kappa B protein alpha; IKK: IκB kinase; JNK: c-Jun N-terminal kinase; M: Molar; MAPK-Mitogen activated protein kinases; min: Minute; mTOR: Mammalian target of rapamycin; NF-κB: Nuclear factor kappa B; PI3-K-phosphatidylinositol-3-kinase; RelA; Reticuloendotheliosis Viral Oncogene Homolog A; RNA: Ribonucleic acid; siRNA: Small interfering RNA; WT: Wild Type; XIAP: X-chromosome linked inhibitor of apoptosis; μM: Micro molar

## Competing interests

The authors declare that they have no competing interests.

## Authors' contributions

All authors read and approved the final manuscript.

VTP and VTC has equally contributed to the *in vitro *viability assays, transfection and NF-κB and AP-1 studies, SVB and RV contributed to the MAPK studies, AKTT carried out the clonogenic assay, BSV and PRP contributed to the detection of expression pattern of p53, IκBα and p-IKK, AB and SV contributed to the expression studies of IAPs and RJA designed and coordinated the study and drafted the manuscript.
